# Sarcopenia and Vitamin B12: Where Do We Stand?

**DOI:** 10.3390/muscles5040069

**Published:** 2026-09-29

**Authors:** Konstantina Mavrou, Nikolaos Ntelis, Nikolaos D. Karakousis, Maria Papagianni

**Affiliations:** Department of Nutrition and Dietetics, School of Physical Education, Sport Science and Dietetics, University of Thessaly, 42100 Trikala, Greece; komavrou@uth.gr (K.M.); nikarakousis@uth.gr (N.D.K.); mpapag@uth.gr (M.P.)

**Keywords:** vitamin B12, sarcopenia, muscle mass, frailty, aging

## Abstract

**Background/Objectives:** Sarcopenia is an age-related skeletal muscle disorder associated with functional decline, frailty, and reduced quality of life. Vitamin B12 plays an essential role in neuromuscular function, DNA synthesis, and homocysteine metabolism, yet its relationship with sarcopenia remains incompletely understood. This narrative review aimed to summarize the current evidence regarding the association between vitamin B12 status and sarcopenia and to discuss the potential biological mechanisms underlying this relationship. **Methods:** A literature search was conducted in PubMed, Scopus, and Web of Science to identify relevant studies published up to 4 September 2026. Twenty studies met the eligibility criteria and were included in this narrative review. Evidence regarding muscle mass, muscle strength, physical performance, and the biological mechanisms linking vitamin B12 to sarcopenia was synthesized narratively. **Results:** The included studies reported heterogeneous findings regarding the association between vitamin B12 status and sarcopenia-related outcomes. Some studies suggested that adequate vitamin B12 status was associated with better muscle mass and physical performance, whereas others found no independent association after adjustment for potential confounding factors. Vitamin B12 deficiency has been linked to neuromuscular dysfunction, hyperhomocysteinemia, and impaired muscle metabolism, providing biological plausibility for a potential association. However, findings regarding muscle strength and the overall association with sarcopenia remained inconsistent across studies, reflecting differences in study populations, diagnostic criteria, and methodological approaches. **Conclusions:** Current evidence suggests heterogeneous and, in some cases, subgroup-specific associations between vitamin B12-related measures and selected sarcopenia-related outcomes. However, methodological heterogeneity, the predominantly observational nature of the evidence, and potential residual confounding preclude causal inferences and do not allow a reliable determination of which individual components of sarcopenia are most consistently associated with vitamin B12.

## 1. Introduction

Vitamin B12, also known as cobalamin, is a water-soluble vitamin that serves as an essential cofactor in several critical metabolic processes, including cellular function, erythropoiesis, neurological maintenance, and DNA synthesis [1]. Vitamin B12 deficiency represents a significant and widespread public health issue. Its etiology is multifactorial, encompassing inadequate dietary intake, genetic predisposition, and acquired conditions that impair B12 absorption, processing, or transport [2]. Among these, age-related malabsorption, poor dietary quality and metabolic changes associated with aging are considered the primary contributing factors [2].

Sarcopenia is defined as a progressive, age-accelerated loss of skeletal muscle mass and function, related to impaired mobility, reduced physical activity and increased risk of adverse outcomes including falls, fractures and premature mortality [3]. Its pathophysiology is multifactorial, involving aging-related hormonal decline, chronic disease, systemic inflammation, oxidative stress, insulin resistance and malnutrition, among other mechanisms [1,3].

Both conditions frequently co-occur in the elderly, who are disproportionately affected by vitamin B12 deficiency due to age-related malabsorption and polypharmacy [1]. One proposed mechanism linked to these two conditions involves homocysteine metabolism. B12 deficiency is associated with elevated serum homocysteine, which has in turn been associated with reduced muscle strength and decreased gait speed, both key components of sarcopenia [1]. Additionally, the role of vitamin B12 in DNA synthesis and amino acid metabolism provides biological plausibility for a potential relationship between B12 status and muscle-related outcomes [4].

Despite extensive research on vitamin B12 in the context of hematopoiesis and neurological function, its independent contribution to the maintenance of muscle mass and strength remains debated [5]. The existing evidence is characterized by methodological heterogeneity and important limitations, underscoring the need for further investigation into the relationship between vitamin B12 status and sarcopenia [6].

### 1.1. Vitamin B12

Vitamin B12 (cobalamin) encompasses several biologically active forms (methylcobalamin, hydroxocobalamin, and adenosylcobalamin), each participating in distinct but interconnected metabolic pathways [1]. Its principal functions include mitochondrial metabolism, DNA biosynthesis and the catabolism of amino acids and fatty acids [1]. Certain cobalamin forms have been proposed to participate in redox-related processes, although the clinical relevance of these mechanisms remains uncertain [5]. Notably, vitamin B12 is indispensable for myelin biosynthesis and maintenance, thereby underpinning peripheral nerve integrity and neuromuscular transmission [7]. Given its involvement in mitochondrial bioenergetics, redox homeostasis, and neuromuscular function, B12 insufficiency has been investigated in relation to muscle strength, physical performance, and mobility, particularly in older adults [8].

Vitamin B12 is found exclusively in foods of animal origin (meat, poultry, fish, eggs, and dairy products), rendering individuals adhering to strict plant-based diets particularly susceptible to deficiency in the absence of fortified foods or supplementation [9]. The European Food Safety Authority (EFSA) recommends a daily intake of 4 μg for adults, 4.5 μg during pregnancy, and 5 μg during lactation, with higher requirements anticipated in older populations due to age-related malabsorption [10].

Intestinal absorption of vitamin B12 is a multistep receptor-mediated process [7]. Following gastric proteolysis, free cobalamin sequentially binds haptocorrin and intrinsic factor (IF) (a glycoprotein secreted by parietal cells) prior to IF-receptor-mediated endocytosis in the distal ileum [8]. Absorbed cobalamin circulates bound to transcobalamin II, with the liver serving as the primary storage depot and site of enterohepatic recirculation [11]. Disruption of gastric acid secretion, pancreatic exocrine malfunction, or ileal integrity, whether through autoimmune atrophic gastritis, pernicious anemia, inflammatory bowel disease, or ileal resection, may substantially impair bioavailability [2]. Prolonged use of proton pump inhibitors, H2-receptor antagonists, or metformin further reduces B12 absorption through pharmacological mechanisms [12].

Vitamin B12 deficiency produces hematological, neurological and metabolic manifestations [13]. Megaloblastic anemia arising from impaired DNA synthesis represents the classical hematological phenotype, whereas neurological sequelae, including peripheral neuropathy, subacute combined degeneration of the spinal cord, and optic neuropathy, may emerge independently of anemia [14]. Additionally, vitamin B12 deficiency is associated with elevated circulating homocysteine and may be accompanied by alterations in oxidative stress, endothelial function, and systemic inflammatory pathways [15].

Serum vitamin B12 is the most widely used diagnostic index, with WHO defining deficiency as concentrations below 148 pmol/L and low-normal status as 148–221 pmol/L [2]. However, serum B12 alone inadequately captures functional intracellular deficiency; concurrent measurement of methylmalonic acid and total homocysteine is therefore recommended to enhance diagnostic specificity [10].

### 1.2. Sarcopenia

Sarcopenia is defined as a progressive skeletal muscle disorder characterized by a systematic decline in muscle mass and physical performance, with predominant manifestation in the elderly [16]. The term is etymologically derived from the Greek words sarx (flesh) and penia (loss/poverty), conceptually reflecting the progressive deterioration of skeletal muscle tissue inherent to the aging process [17]. This condition has been increasingly recognized as a significant public health issue, given its well-established associations with disability, frailty, fractures, hospitalization, impaired quality of life, institutionalization, and premature mortality [17].

In accordance with the revised diagnostic framework proposed by the European Working Group on Sarcopenia in Older People 2 (EWGSOP2), sarcopenia is stratified into three distinct stages: probable sarcopenia, confirmed sarcopenia and severe sarcopenia [16]. Reduced muscle strength constitutes the primary determinant for the identification of probable sarcopenia, whereas concurrent documentation of low muscle quantity or quality confirms the diagnosis [16]. Severe sarcopenia is established upon the additional presence of impaired physical performance [16]. This updated classification emphasizes that muscle strength and functional performance demonstrate greater clinical relevance as predictors of adverse outcomes than muscle mass quantification alone [16].

The diagnostic evaluation of sarcopenia encompasses the systematic assessment of muscle strength, physical performance and the estimation of decline in muscle quantity and quality [17]. Handgrip strength is widely employed as a quite reliable and clinically practicable surrogate measure of overall muscle strength [17]. Changes in muscle quantity and quality are predicted by imaging and body composition methodologies, including dual-energy X-ray absorptiometry (DXA), magnetic resonance imaging (MRI), computed tomography (CT), ultrasonography, and bioelectrical impedance analysis (BIA) [18]. Although BIA is frequently utilized due to its accessibility and cost-effectiveness, its diagnostic accuracy might be compromised by variations in hydration status and body composition [19]. Anthropometric prediction equations have also been proposed for estimating skeletal muscle mass when imaging techniques are unavailable. One of the most widely used models is the equation developed by Lee et al. [20], which estimates skeletal muscle mass using anthropometric variables including body weight, height, age, sex, and ethnicity. This equation was developed and cross-validated against magnetic resonance imaging (MRI) and has been extensively applied in epidemiological studies due to its practicality and acceptable validity [20]. Physical performance is conventionally evaluated through gait speed, the Short Physical Performance Battery (SPPB), and the Timed Up and Go (TUG) test, with gait speed recognized as one of the most robust independent predictors of functional decline and all-cause mortality in older adults [17].

The prevalence of sarcopenia exhibits substantial heterogeneity across populations and geographical regions, attributable to differences in age distribution, sex, ethnicity, lifestyle behaviors, comorbidity burden, and the application of varying diagnostic criteria [21]. Whilst advancing age represents the most consistently identified risk factor, the pathophysiology of sarcopenia is inherently multifactorial, encompassing chronic low-grade inflammation, oxidative stress, mitochondrial dysfunction, hormonal dysregulation, neuromuscular degeneration, physical inactivity, and inadequate nutritional intake [22].

Primary sarcopenia is attributed to the physiological aging process itself, whereas secondary sarcopenia may develop in the context of chronic disease states, malnutrition, obesity, prolonged immobilization, or systemic inflammatory conditions. Individuals with chronic diseases demonstrate significantly elevated rates of sarcopenia relative to healthy reference populations [21]. Furthermore, suboptimal dietary protein intake and micronutrient deficiencies may accelerate skeletal muscle deterioration and compromise physical performance, particularly among frail older adults [23].

Sarcopenia carries substantial clinical and functional implications that adversely affect mobility, independence, and overall health-related quality of life [24]. Reduction in muscle mass and strength contribute to impaired physical performance, elevated risk of falls and fragility fractures, functional disability, acute hospitalization, and long-term institutionalization [25]. In addition, sarcopenia has been independently related to increased healthcare resource utilization, prolonged inpatient stays, depressive symptomatology, cognitive decline, social isolation, and elevated all-cause mortality risk among community-dwelling older individuals [25].

Accumulating evidence suggests that nutritional factors may be associated with sarcopenia and its related outcomes [26]. Among these, vitamin B12 has garnered increasing scientific interest owing to its involvement in mitochondrial metabolism, DNA synthesis, neuromuscular function, and homocysteine metabolism [27]. Vitamin B12 deficiency has been associated with several biological alterations, including peripheral neuropathy, oxidative stress, mitochondrial dysfunction, altered energy metabolism, and elevated circulating homocysteine levels, which may provide biological plausibility for a potential relationship with sarcopenia-related outcomes [28].

Neurological manifestations related to vitamin B12 insufficiency, including gait disturbances, balance impairment, generalized muscle weakness, sensory deficits, and reduced physical performance, may further exacerbate functional decline and frailty in aging populations [26]. Elevated homocysteine concentrations have additionally been associated with endothelial dysfunction, chronic inflammation, and impaired skeletal muscle function, providing biological plausibility for a potential association between suboptimal vitamin B12 status and sarcopenia-related outcomes [29].

Although currently available evidence remains largely heterogeneous and predominantly observational in nature, the reported associations between vitamin B12 status, frailty, neuromuscular function, and physical performance support further investigation of the relationship between vitamin B12 status and skeletal muscle health in aging populations.

Nevertheless, several mechanistically plausible pathways may provide biological plausibility for a potential relationship between B12 insufficiency and sarcopenia-related outcomes [19]. Proposed pathways include neuromuscular dysfunction related to demyelination, mitochondrial alterations, homocysteine-related metabolic disturbances, and oxidative stress, although the extent to which these mechanisms contribute independently to sarcopenia remains uncertain [20]. Older individuals have a higher prevalence of vitamin B12 deficiency and are also at increased risk of sarcopenia [21].

The aim of the present narrative non-systematic review is to explore the potential association between vitamin B12 status and sarcopenia and its related components. Given the predominantly observational and heterogeneous nature of the available evidence, particular attention is given to the consistency of reported associations and the limitations affecting causal interpretation [5].

## 2. Materials and Methods

**Figure 1:**
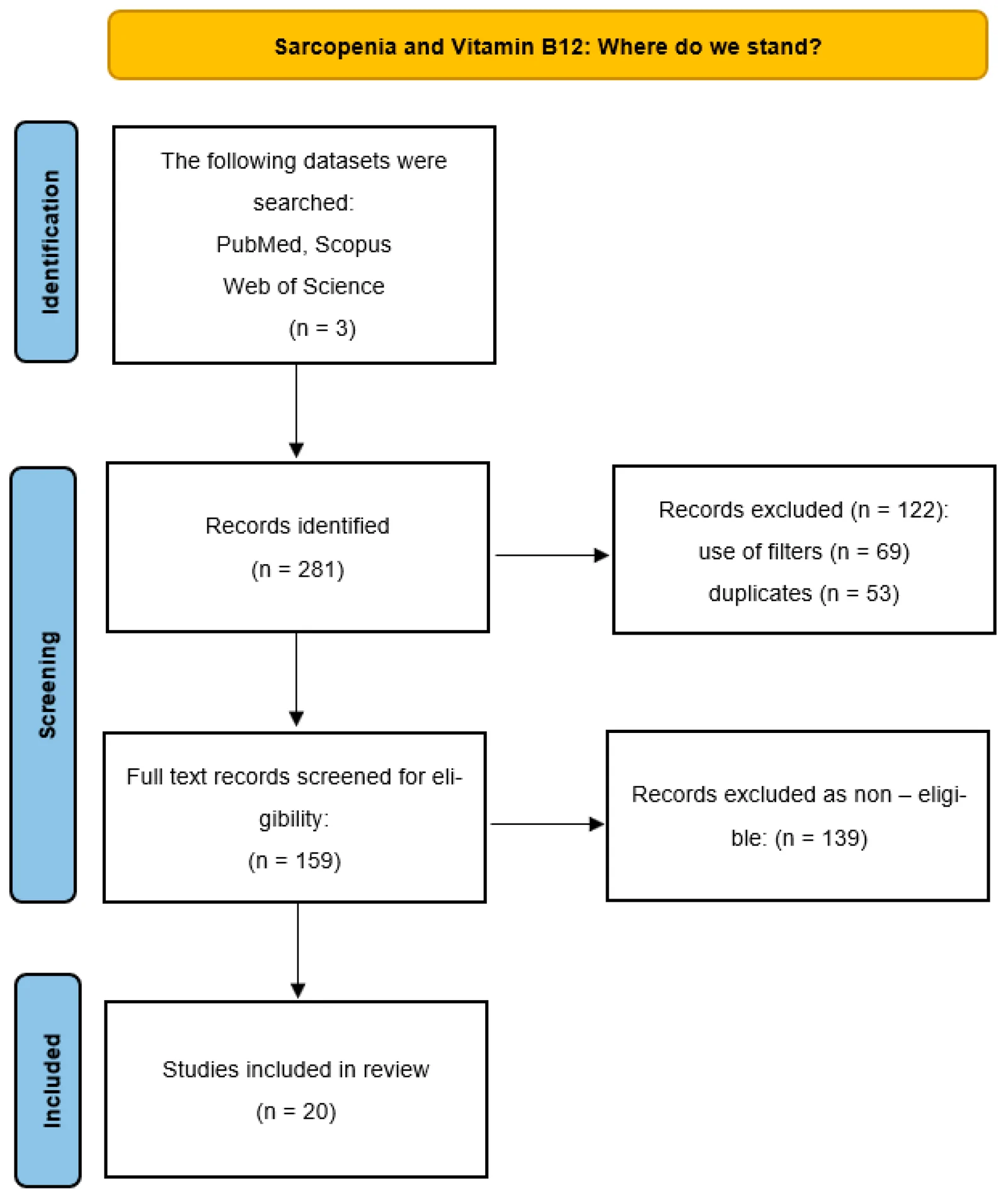
Study selection flowchart concerning vitamin B12 and sarcopenia association.

In this narrative non-systematic review, a structured search was performed across three major electronic databases—PubMed, Scopus, and Web of Science—to identify studies examining the interplay between vitamin B12 status and sarcopenia. The final database search was performed on 4 September 2026. The search strategy employed the following keyword combinations: “Vitamin B12 or cobalamin and sarcopenia,” “Vitamin B12 or cobalamin and low muscle mass,” “Vitamin B12 or cobalamin and low muscle strength,” and “Vitamin B12 or cobalamin and muscle performance.” The database-specific search strategies are provided in Supplementary Material S1. To identify clinically relevant studies addressing the association between vitamin B12 and sarcopenia, predefined filters were applied prior to screening. Studies published between January 2010 and 4 September 2026 were considered, reflecting the growing research interest in micronutrient–muscle interactions over the past decade. Studies were eligible for inclusion if they were original research articles, written in English, involved human participants, and directly investigated the association between vitamin B12 and sarcopenia or its diagnostic components (muscle mass, muscle strength, or physical performance). Studies were excluded if they were conducted in animals or in vitro, did not directly address the vitamin B12–sarcopenia interplay, were published in languages other than English, or were classified as reviews or case reports. Duplicate records were removed before title/abstract screening. The database search initially identified 281 records. Following application of the predefined filters, 212 records remained. After removal of 53 duplicate records, 159 records underwent title/abstract screening. Of these, 139 records were excluded because they did not meet the predefined eligibility criteria. The remaining 20 articles were subsequently assessed in full text against the predefined eligibility criteria. All 20 articles met the eligibility criteria following full-text assessment and were included in the final narrative synthesis. The study-selection process is illustrated in the study-selection flowchart (Figure 1).

## 3. Results

The outcomes of this non-systematic review study are depicted in Table 1.

### 3.1. Vitamin B12 Levels and Outcomes Assessed

Vitamin B12 plays a fundamental role in the synthesis and maintenance of myelin, thereby preserving neurological integrity and neuromuscular function [1]. Deficiency of vitamin B12 may lead to neurological manifestations, including impaired balance, gait disturbances, peripheral neuropathy, and muscle weakness, all of which may contribute to the development or progression of sarcopenia [32].

Zhao et al. [30] in a cross-sectional study, investigated the association between vitamin B12 status and musculoskeletal health using data from the National Health and Nutrition Examination Survey (NHANES) in adults aged over 50 years [30]. Among 11,039 initial participants, 1466 completed dual-energy X-ray absorptiometry (DXA) measurements and functional performance tests. Serum concentrations of vitamin B12 and plasma homocysteine, and methylmalonic acid were measured alongside indicators of muscle mass, gait speed, and knee extensor strength. The study demonstrated that lower serum vitamin B12 concentrations were associated with elevated homocysteine and methylmalonic acid plasma levels (*p* < 0.05) [30]. Higher homocysteine concentrations were further correlated with reduced lean body mass, slower gait speed, and decreased knee extensor strength in older adults (*p* < 0.05) [30]. However, after adjustment for potential confounding variables, the associations between vitamin B12 levels and the assessed measures of muscle mass and physical function were no longer statistically significant in individuals over 50 years of age [30]. In the subgroup of participants aged ≥65 years, serum vitamin B12 was positively associated with total lean mass (*p* = 0.044) and appendicular lean mass (*p* = 0.043) after full adjustment [30].

Van Schoor et al. [41] investigated the relationship between plasma homocysteine, serum vitamin B12, and physical performance using both cross-sectional and longitudinal analyses in adults aged ≥65 years [41]. After adjustment for potential confounders, higher plasma homocysteine concentrations were associated with lower physical performance among women in both the cross-sectional (*p* < 0.01) and longitudinal analyses (*p* = 0.05). In contrast, associations between serum vitamin B12 concentrations and physical performance were less consistent [41].

Wolffenbuttel et al. [45] evaluated serum vitamin B12, methylmalonic acid (MMA), and muscle strength in 9645 NHANES participants [45]. Serum vitamin B12 was not independently associated with muscle strength in multivariable analysis (*p* = 0.046; predefined significance threshold *p* < 0.01). In contrast, higher MMA concentrations were independently associated with lower muscle strength (*p* < 0.001) [45].

Chae et al. [32], using data from the Korean Frailty and Aging Cohort Study (KFACS), evaluated 2325 community-dwelling older adults and examined the association between serum vitamin B12 concentrations and sarcopenia-related parameters [32]. Vitamin B12 deficiency was associated with lower muscle mass (*p* = 0.002) after adjustment for potential confounding variables. However, no statistically significant associations were identified between vitamin B12 status and muscle strength or physical performance [32]. These findings indicate an association between vitamin B12 status and muscle mass, but do not establish a causal effect given the cross-sectional study design.

Similarly, Ates Bulut et al. [5] conducted a prospective study including 403 older adults with and without sarcopenia. Lower vitamin B12 levels were associated with lower muscle mass and muscle strength (*p* < 0.05) [5]. The frequency of sarcopenia and dynapenia was higher among subjects with vitamin B12 deficiency (*p* < 0.05) [5].

Scott et al. [37] conducted a cross-sectional study including 1576 adults from the Baltimore Longitudinal Study of Aging and examined the association between vitamin B12 levels and appendicular skeletal muscle mass [37]. In women, higher vitamin B12 levels were positively associated with appendicular skeletal muscle mass (*p* = 0.035), after adjustment for height [37]. No statistically significant associations were observed between vitamin B12 levels and muscle strength or physical functionality [37].

Although Ao et al. [40] in a cross-sectional study, show a non-significant association between serum vitamin B12 with lower muscle strength and physical endurance, in 65 women over 65 years old [40]. The study initially enrolled 86 participants. However, only 77 participants completed the more detailed assessments for safety reasons. Of these, only the 65 female participants were included in the final analyses because women constituted most of the study population [40].

Soh et al. [33] examined the association between vitamin B12 status, frailty, and physical performance in older adults. In the unadjusted analysis, vitamin B12 insufficiency was associated with a higher prevalence of frailty (*p* = 0.038). However, this association was attenuated and was no longer statistically significant after multivariable adjustment. Similarly, differences in several physical-performance measures observed in the unadjusted analyses were no longer statistically significant after full adjustment. In contrast, the association with 4 -mgait speed remained statistically significant after full multivariable adjustment. Overall, the findings did not provide consistent evidence of an independent association between vitamin B12 status and frailty or physical performance after adjustment [33].

Similarly, Wee et al. [39] assessed muscle strength and physical performance in 56 older diabetic adults, in a cross-sectional study [39]. The researchers found no statistically significant association between vitamin B12 concentrations and reduced muscle strength or impaired physical performance (*p* > 0.05). Nevertheless, vitamin B12 deficiency may still contribute indirectly to musculoskeletal dysfunction through neuropathy, impaired balance, and gait instability [39].

Finally, Pedrero-Chamizo et al. [43] longitudinally evaluated serum vitamin B12 status and functional markers in 60 institutionalized older adults aged 62–99 years. Serum cobalamin concentrations decreased during follow-up (299 to 273 pmol/L, *p* < 0.05) [43]. However, serum cobalamin was not significantly associated with handgrip, arm, or leg strength in the linear mixed models [43].

### 3.2. Vitamin B12 Intake and Outcomes Assessed

Additional nutritional studies support the potential contribution of vitamin B12 to muscle health. Borda et al. [35] conducted a cross-sectional study, about nutrient intake in association with sarcopenia markers [35]. Borda et al. collected data from the Gothenburg H70 birth cohort study in Sweden and performed screening in 719 subjects of old age, identifying positive associations between vitamin B12 intake and appendicular lean mass (*p* < 0.001) [35].

Furthermore, Polo-Ferrero et al. [36] conducted a secondary observational analysis within a randomized exercise intervention trial including 80 older adults at high risk of sarcopenia. Vitamin B12 intake was assessed as an observational dietary exposure, rather than as a randomized intervention. Higher vitamin B12 intake was associated with better muscle performance among women, in both exercise interventions (*p* < 0.05) [36].

Takahashi et al. [34] conducted a prospective study of 197 older adults with type 2 diabetes in Japan to examine the association between vitamin B12 intake and changes in muscle mass during follow-up. Lower vitamin B12 intake was barely statistically significant associated with greater muscle mass loss (*p* = 0.049), after adjustment for relevant confounding variables [34]. In subgroup analyses, vitamin B12 intake was also associated with lower muscle mass loss among participants with low energy intake, those using supplements, and those not receiving antidiabetic treatment (*p* = 0.013) [34].

Gedmantaite et al. [42] examined the association between dietary vitamin B12 intake and handgrip strength in 68,002 UK Biobank participants [42]. In sex-stratified adjusted analyses, higher vitamin B12 intake was positively associated with handgrip strength in women (*p* < 0.0001). In men, the corresponding association (*p* = 0.014) did not meet the Bonferroni-adjusted significance threshold (*p* < 0.0019) [42].

Finally, Jia et al. [38] conducted a cross-sectional study including 3648 obese and non-obese older adults from the NHANES study. In obese women, higher vitamin B12 intake was associated with a higher likelihood of low muscle mass (*p* = 0.014), whereas no significant association was observed in the other groups [38].

### 3.3. Vitamin B12 and Sarcopenia

Cobalamin is a vitamin involved in several metabolic pathways, thereby contributing to neuromuscular and musculoskeletal health [1]. Sarcopenia is a multifactorial syndrome characterized by progressive loss of skeletal muscle mass and physical performance, and is associated with aging, chronic diseases, inflammation, oxidative stress, and nutritional deficiencies [38].

Choi et al. [6] conducted a longitudinal analysis of 844 older adults without baseline sarcopenia using KFACS data [6]. During a two-year follow-up period, all sarcopenia-related parameters declined; however, in a fully adjusted analyses, women with vitamin B12 deficiency exhibited significantly greater deterioration in physical performance and higher incidence of sarcopenia compared with women with adequate vitamin B12 levels (*p* < 0.05) [6]. In contrast, no statistically significant associations were identified in men [6].

Petermann-Rocha et al. [44] examined factors associated with sarcopenia, defined according to the EWGSOP2 criteria, in 396,283 UK Biobank participants [44]. Higher dietary vitamin B12 intake was associated with lower odds of sarcopenia, with participants in the highest tertile of vitamin B12 intake having lower odds compared with those in the lowest tertile (adjusted *p* < 0.001) [44].

In addition, Verlaan et al. [4] conducted a patient-control study, including 66 sarcopenic and 66 non-sarcopenic patients [4]. Sarcopenic patients, evaluated with SPPB score and SMI score, had lower intakes of vitamin B12 in contrast with non-sarcopenic patients (*p* = 0.011) [4]. Vitamin B12 deficiency was also more frequent among sarcopenic patients (*p* = 0.033) [4].

Van Atteveld et al. [46] investigated sarcopenia in adult survivors of childhood cancer using the EWGSOP2 definition [46]. Among participants with complete sarcopenia measurements (n = 1472), vitamin B12 deficiency was independently associated with substantially higher odds of sarcopenia (multivariable adjusted *p*= 0.0007) [46].

Finally, Liu et al. [31] examined the association between vitamin intake and the risk of sarcopenia in 5256 subjects with a mean age of 36 years. Higher vitamin B12 intake was associated with a lower risk of sarcopenia in middle-aged women (*p* = 0.008) and with higher muscle mass in the overall sample (*p* < 0.05) [31].

## 4. Discussion

This review examined the potential association between vitamin B12 status and sarcopenia, incorporating studies that investigated vitamin B12-related measures in relation to muscle mass, muscle strength, physical performance, and sarcopenia-related outcomes [4,5,6]. The available evidence suggests that vitamin B12-related measures may be associated with certain components of sarcopenia; however, the findings remain inconsistent and do not establish an independent causal or protective effect [30,31].

Vitamin B12 deficiency has been linked to a range of neurological consequences, including demyelination, myelopathy, and peripheral neuropathy, all of which may impair motor, sensory, and cognitive function [6]. At the metabolic level, inadequate vitamin B12 status is associated with elevated homocysteine concentrations, which have been associated with alterations in skeletal muscle through pathways involving oxidative stress, systemic inflammation, and microvascular function [30]. Beyond its neurological and vascular effects, vitamin B12 deficiency may be relevant to pathways involved in muscle maintenance and regeneration [6]. Vitamin B12 is an essential cofactor in one-carbon metabolism and cellular DNA synthesis and may indirectly influence muscle health through metabolic and neurological pathways [5]. Moreover, vitamin B12 deficiency has been associated with depression and cognitive decline, which may in turn be related to physical inactivity and reduced social engagement, particularly in older adults [32]. Collectively, these mechanisms provide biological plausibility for a potential relationship between vitamin B12 deficiency and sarcopenia-related outcomes; however, they do not establish a direct or independent causal effect [47].

At the nutritional level, B-complex vitamins, and vitamin B12 in particular, have been associated with a reduced likelihood of low muscle mass [48]. In adults over 50 years of age, higher vitamin B12 intake has been associated with a lower prevalence of sarcopenia [48]. Consistent with these observations, higher dietary vitamin B12 intake was associated with lower odds of sarcopenia in a large UK Biobank population [44], while vitamin B12 deficiency was independently associated with higher odds of sarcopenia among adult childhood cancer survivors [46]. Higher dietary vitamin B12 intake was also positively associated with handgrip strength among women in the UK Biobank [42].

However, findings for muscle strength and physical performance were less consistent. Other studies did not demonstrate consistent independent associations between serum vitamin B12 concentrations and muscle strength or physical performance [43,45]. Notably, functional outcomes appeared to show stronger associations with the vitamin B12-related biomarkers homocysteine and methylmalonic acid than with serum vitamin B12 itself [41,45]. Vitamin B12 supplementation has been shown to reduce plasma homocysteine and methylmalonic acid concentrations; however, whether these biochemical effects translate into improvements in muscle mass, strength, physical performance, or sarcopenia remains uncertain [3,47]. Homocysteine and methylmalonic acid are indirect biomarkers of vitamin B12 status and may also be influenced by factors unrelated to vitamin B12; therefore, findings based on these biomarkers should be interpreted cautiously [48].

Taken together, these findings support a potential association between vitamin B12-related measures and selected sarcopenia-related outcomes, while also emphasizing substantial heterogeneity according to the vitamin B12 measure, outcome assessed, study population, and study design. Overall, the current evidence regarding the association between vitamin B12 status and multiple components of sarcopenia remains inconsistent, and causality cannot yet be established due to the predominantly observational nature of the available studies and the substantial methodological heterogeneity. Further large-scale prospective cohort studies and randomized controlled trials are required to clarify the underlying biological mechanisms and determine whether vitamin B12 status has an independent and clinically meaningful relationship with sarcopenia-related outcomes.

The present narrative non-systematic review is subject to several limitations that should be considered when interpreting its findings. The majority of included studies employed observational designs, which inherently preclude the establishment of causal relationships between vitamin B12 status and sarcopenia. Randomized controlled trials are needed to determine whether vitamin B12 supplementation has an independent effect on muscle mass, strength, physical performance, or sarcopenia risk. Considerable heterogeneity was observed across studies in terms of sample size, population characteristics, and generalizability. Several studies were conducted in small or highly specific samples, predominantly older adults, a single sex, or populations with particular health conditions, limiting the applicability of findings to the broader general population.

Methodological inconsistency across studies represents an additional limitation. Different sarcopenia diagnostic criteria and assessment tools were applied, and the thresholds used to define vitamin B12 deficiency and sufficiency varied considerably between studies, making direct comparisons difficult and contributing to heterogeneity in reported outcomes. Furthermore, most studies examined the role of micronutrients in sarcopenia broadly rather than focusing specifically on vitamin B12, and the relatively small number of studies directly addressing this relationship constrains the strength of the conclusions that can be drawn.

The potential influence of confounding factors also cannot be fully excluded. Overall nutritional status and diet quality, protein and energy intake, comorbidities, renal function, medication use, physical activity, socioeconomic factors, and the possibility of reverse causation may all have affected the reported outcomes. Incomplete adjustment for these variables may have led to either overestimation or underestimation of the association between vitamin B12 and sarcopenia. Therefore, the findings should be interpreted with caution, as these factors may have contributed to the observed associations independently of vitamin B12 status.

An additional consideration is the potential overlap in study populations among the included publications. Several studies, including those by Chae et al. [32], Soh et al. [33], and Choi et al. [6], used data from the Korean Frailty and Aging Cohort Study (KFACS). Although these studies examined different outcomes and, in the case of Choi et al., longitudinal follow-up, their findings cannot be considered fully independent replications because of the underlying cohort overlap. Accordingly, the consistency of the evidence should be interpreted at the level of distinct cohorts rather than by simply counting the number of publications reporting an association. This potential non-independence further limits the strength of conclusions regarding the consistency of the association between vitamin B12-related measures and muscle-related outcomes.

Lastly, as this was a narrative review, no formal risk-of-bias or methodological quality assessment was performed. Therefore, the findings should be interpreted with caution, considering the heterogeneity of the included study designs and the absence of a standardized critical appraisal. Taken together, these limitations highlight several priorities for future research.

Studies with more diverse samples are imperative, encompassing different age groups, sexes, and socioeconomic backgrounds, in order to improve the generalizability of findings to the broader population. To date, the majority of available studies have focused on specific subgroups, constraining the external validity of their conclusions. From a methodological standpoint, future research should prioritize study designs with greater scientific rigor. In particular, randomized controlled trials are warranted to determine whether vitamin B12 supplementation affects muscle mass, strength, physical performance, or sarcopenia risk. Complementing this, longitudinal studies with extended follow-up periods are essential to evaluate the trajectory of sarcopenia in relation to vitamin B12 status over time.

Standardization of both sarcopenia diagnostic criteria and vitamin B12 sufficiency thresholds represents a critical priority for the field. The adoption of uniform assessment tools and common cut-off values would minimize heterogeneity across studies, facilitate meaningful comparisons, improve the reliability of conclusions, and reduce the risk of misclassification. Future studies should also ensure rigorous adjustment for confounding variables, including physical activity, overall diet quality, comorbidities, and medication use, to allow for a more precise estimation of the independent association between vitamin B12-related measures and sarcopenia-related outcomes. Investigating the interaction between vitamin B12 and other nutritional or metabolic factors may further deepen our understanding of the underlying mechanisms.

Finally, future research should determine whether vitamin B12 status or supplementation has a clinically meaningful role in sarcopenia prevention or management, particularly in populations at increased risk. Establishing such effects through appropriately designed prospective and interventional studies would be necessary before clinical recommendations regarding vitamin B12 supplementation for sarcopenia prevention or management can be made.

From a clinical perspective, screening for vitamin B12 deficiency may be considered in individuals at increased risk of deficiency, particularly older adults with poor dietary intake, malabsorption disorders, long-term use of medications that may impair vitamin B12 absorption, or clinical manifestations compatible with vitamin B12 deficiency. Assessment may also be considered in older adults presenting with unexplained muscle weakness, impaired physical performance, or other features suggestive of sarcopenia, particularly when additional risk factors for vitamin B12 deficiency are present. However, the current evidence does not support universal vitamin B12 screening specifically for the prevention or management of sarcopenia, and further prospective studies are required to determine whether targeted screening and correction of vitamin B12 deficiency improve musculoskeletal outcomes.

## 5. Conclusions

This narrative non-systematic review examined the available evidence regarding the association between vitamin B12-related measures and selected sarcopenia-related outcomes. Overall, the findings were heterogeneous and, in some cases, subgroup-specific. Consequently, an independent causal relationship between vitamin B12 status and sarcopenia cannot currently be established. Vitamin B12 deficiency is known to affect neurological function, one-carbon metabolism, and hematological processes, providing biological plausibility for a potential association with muscle-related outcomes. However, whether optimization of vitamin B12 status independently influences muscle mass, muscle strength, physical performance, or sarcopenia risk remains uncertain. Further well-designed prospective studies and randomized controlled trials are required to determine whether vitamin B12 status has an independent effect on muscle mass, muscle strength, physical performance, or sarcopenia risk.

## Figures and Tables

**Table 1:** The interplay between sarcopenia and vitamin B12.

Authors/ [Reference]	Study Design	Study Population and Characteristics	Vitamin Β12—Related Measure/Exposure	Outcome Assessed	Sarcopenia Definition	Main Outcomes
Choi et al. 2023 [6]	Prospective study	844 subjects. 382 men and 462 women. Age 76 ± 3.9 years without sarcopenia	B12 levels	Sarcopenia	AWGS 2019	Vitamin B12 deficiency was associated with low physical performance. OR = 4.38, (95% confidence interval [CI] = 2.15–8.84) (*p* < 0.001) and high risk for sarcopenia, only in women OR = 5.90, (95% CI = 1.55–22.43) (*p* < 0.05) fully adjusted.
Ates Bulut et al. 2017 [5]	Prospective study	403 subjects. Age 77.8 ± 5.76 years with Sarcopenia and age 71.8 ± 7.43 years without sarcopenia	B12 levels	Muscle mass Muscle strength	EWGSOP criteria	Low vitamin B12 levels were associated with low skeletal muscle mass (mean ± SD: 26.01 ± 4.51 kg) and muscle strength (17.95 ± 9.06 kg) (*p* < 0.05) in sarcopenic vs. non-sarcopenic subjects.
Verlaan et al. 2017 [4]	Case–control study	132 subjects. Age 71 ± 4 years 66 with sarcopenia and 66 without sarcopenia	Dietary B12 intake; B12 levels	Sarcopenia	SPPB 4–9 and low SMI	Sarcopenic patients had lower intake of vitamin B12 (median (IQR): 3.9 (2.6, 5.9)) in comparison with the patients with no sarcopenia. 5.3 (3.6, 6.9) (*p* = 0.011). Serum vitamin B12 concentrations were also lower in sarcopenic participants (284 ± 107 vs. 335 ± 120 pmol/L, *p* = 0.015), and vitamin B12 deficiency was more frequent (26.2% vs. 10.9%, *p* = 0.033)
Zhao et al. 2024 [30]	Cross-sectional	1466 adults. Age ≥ 50 years Subgroup analysis Age ≥ 65 years	B12 serum levels, Hcy and MMA levels	Total lean mass Appendicular skeletal muscle mass	NA	In fully adjusted analyses, serum vitamin B12 was not significantly associated with muscle-related outcomes in the overall population (*p* > 0.05). In participants aged ≥65 years, serum vitamin B12 was positively correlated with total free fat mass and appendicular skeletal muscle mass; β coefficient ± 95% CL: (β = 584.83 ± 14.84, 1154.73 *p* = 0.044; β= 291.65 ± 8.68, 574.60 *p* = 0.043) both adjusted for age, sex, race, smoking status, alcohol intake, height, fat mass (%), physical activity, cardiovascular disease, diabetes, cancer, liver impairment, renal impairment, serum folate, serum 25(OH) D, total serum calcium and vitamin K intake.
Liu et al. 2024 [31]	Cross-sectional	5256 subjects. Age 36 ± 14.1 year	B12 intake	Sarcopenia	FNIH	High vitamin B12 intake was associated with low risk of sarcopenia in mid age women OR (%95CI) 0.293 (0.133–0.649) (*p* = 0.008) adjusted for sex, age, race, BMI, education level, physical activity, smoking status, drinking history, hypertension, diabetes, cardiovascular disease, and cancer.
Chae et al. 2021 [32]	Cross-sectional	2325 subjects 76.4 ± 3.6 years	B12 levels	Muscle mass	NA	The group with vitamin B12 deficiency had lower muscle mass than the group with normal vitamin B12 levels. OR %95 CI: 1.478 (1.155, 1.891). *p* = 0.002 adjusted for age, sex, depression, osteoarthritis, osteoporosis, diabetes mellitus, hypertension, smoking, alcohol consumption, location of residence, and body mass index.
Soh et al. 2020 [33]	Cross-sectional	2938 subjects. Age 76.4 ± 3.8 years	B12 levels	Frailty; Physical performance	NA	In unadjusted analyses, vitamin B12 insufficiency was associated with higher frailty prevalence (OR = 1.298, 95% CI 1.014–1.661, *p* = 0.038). The association was attenuated and no longer statistically significant after multivariable adjustment. Several physical-performance measures showed differences in unadjusted analyses that were not maintained after full adjustment; gait speed showed a distinct pattern and was therefore considered separately. No consistent independent association was observed after adjustment.
Takahashi et al. 2021 [34]	Prospective study	197 Adults with type II diabetes. Age ≥ 65 years	B12 intake	Muscle mass	NA	High vitamin B12 intake was associated with lower risk of low muscle mass OR (95% CI): 0.92 (0.84–1.00), *p* = 0.049 adjusted for sex, age, insulin treatment, body mass index, skeletal muscle mass index, smoking habit, exercise habit, energy intake, protein intake, habitual alcohol consumption. In subgroups with low energy intake, supplement use and no use of antidiabetic treatment, vitamin B12 supplementation was associated with lower loss of muscle mass OR (95% CI) 0.88 (0.79–0.97), *p* = 0.013 adjusted for sex, age, body mass index, energy intake, and protein intake.
Borda et al. 2024 [35]	Cross-sectional	719 subjects. Age 70.54 ± 0.26 years	B12 intake	Appendicular lean soft tissue	NA	High vitamin B12 intake was associated with higher appendicular lean soft tissue. Mean (SE) = 0.0397 (0.0104) (*p* < 0.001 adjusted).
Polo-Ferrero et al. 2025 [36]	Secondary observational analysis within an RCT (exercise intervention)	80 subjects with elevated risk of sarcopenia. Age ≥ 65 years	B12 intake	Muscle performance	NA	Women with higher vitamin B12 intake had better muscle performance compared to women with low vitamin B12 intake [(mean ± SD) H-RT group; Low performance = 7.6 ± 1.6. High performance = 12.2 ± 2.5 *p* = 0.028; MT group; Low performance = 7.1 ± 1.7. High performance = 12.2 ± 1.7 *p* = 0.006]
Scott et al. 2025 [37]	Cross-sectional	1576 subjects, adults	B12 levels	Appendicular skeletal muscle mass	NA	In women, high levels of vitamin B12 were associated with appendicular skeletal muscle mass. (β coefficient ± Standard error: 0.00017 ± 0.00008) (*p* = 0.035) adjusted for height. Vitamin B12 was not associated with muscle strength and functionality.
Jia et al. 2023 [38]	Cross-sectional	3648 subjects. 1748 men and 1900 women. Obese and non-obese. Men’s age: 65–74 years = 1212, >75 years = 536 Women’s age: 65–74 years = 1246, >75 years = 654	B12 intake	Muscle mass	NA	In obese women only, higher vitamin B12 intake was associated with high risk of lower muscle mass. OR CI%95: 1.169 (1.033, 1.322) (*p* = 0.014) adjusted for poverty/income ratio, education, smoking, drinking, physical activity, comorbidities (hypertension, hyperlipidemia, diabetes, CVD, CRD, and CKD), other dietary nutrients (protein, carbohydrate, fat and fiber) and total energy intake. No correlation between vitamin B12 and sarcopenia or muscle mass in other groups.
Wee et al. 2016 [39]	Cross-sectional	56 patients with diabetes mellitus type II. Men’s age 72 ± 5 years, women’s age 71 ± 4 years	B12 levels	Muscle strength	NA	No correlations between vitamin B12 levels and muscle strength (*p* > 0.05)
Ao et al. 2019 [40]	Cross-sectional	65 subjects, women. Age 84 ± 7.1 years	B12 levels	lower limb muscle strength handgrip strength	NA	Non-significant correlation between vitamin B12 and lower limb muscle strength (adjusted *p* = 0.626) and handgrip strength (adjusted *p* = 0.662)
van Schoor et al., 2012 [41]	Cross-sectional	Adults ≥ 65 years	B12 levels Plasma Hcy	Physical performance	NA	Higher homocysteine was associated with lower physical performance in women subgroup (β = −0.93, SE = 0.34, *p* < 0.01; longitudinal: β = −0.69, SE = 0.35, *p* = 0.05) adjusted for age, region, body mass index, smoking, alcohol use and serum 25-hydroxyvitamin D. Associations between vitamin B12 serum levels and physical performance were less consistent.
Gedmantaite et al., 2020 [42]	Cross-sectional	68,002 UK Biobank participants; age 63.8 ± 2.7 years; 50.3% women	B12 intake	Handgrip strength	NA	Higher vitamin B12 intake was positively associated with handgrip strength in women (standardized β ≈ 0.13, 95% CI ≈ 0.07–0.19, *p* < 0.0001; adjusted analysis for age, month of assessment, ethnicity, deprivation, height, comorbidities and total energy intake), whereas the association in men did not meet the Bonferroni-adjusted significance threshold (*p* = 0.014; significance threshold *p* < 0.0019)
Pedrero-Chamizo et al., 2020 [43]	Longitudinal	60 institutionalized adults aged 62–99 years; 41 women/19 men	B12 levels	Handgrip, arm and leg strength	NA	Serum cobalamin decreased during follow-up (299 to 273 pmol/L, *p* < 0.05); however, serum cobalamin was not significantly associated with handgrip, arm, or leg strength in the linear mixed models.
Petermann-Rocha et al., 2020 [44]	Cross-sectional	396,283 UK Biobank participants; 38–73 years; 52.8% women	B12 intake	Sarcopenia	EWGSOP2	Higher self-reported dietary vitamin B12 intake was associated with lower odds of sarcopenia (middle vs. lowest tertile: adjusted OR = 0.81, 95% CI 0.67–0.97, *p* = 0.026; highest vs. lowest tertile: adjusted OR = 0.66, 95% CI 0.54–0.80, *p* < 0.001).
Wolffenbuttel et al., 2020 [45]	Cross-sectional	9645 NHANES participants > 19 years; age 49 ± 17 years; 49.3% men	B12 levels MMA levels	Handgrip strength	NA	Serum vitamin B12 was not independently associated with muscle strength, whereas higher MMA was independently associated with lower muscle strength. In multivariable analysis, log serum B12: coefficient = −1.275, SE = 0.615, *p* = 0.046; log MMA: coefficient = −4.414, SE = 0.607, *p* < 0.001 (predefined significance threshold *p* < 0.01).
van Atteveld et al., 2023 [46]	Cross-sectional	Adult childhood cancer survivors; 2003 participants overall; complete sarcopenia measurements n = 1472; mean age ≈33 years	B12 levels	Sarcopenia	EWGSOP2	Vitamin B12 deficiency was independently associated with higher odds of sarcopenia (multivariable OR = 6.26, 95% CI 2.17–18.1, *p* = 0.0007 adjusted for male sex, lower BMI, cranial irradiation, total body irradiation Hypogonadism, growth hormone deficiency, vitamin B12 deficiency).

Abbreviations: Hcy: Homocysteine; MMA: methylmalonic acid; AWGS: Asian Working Group for Sarcopenia; SPPB: short physical performance battery; SMI: skeletal muscle mass index; FNIH: Foundation for the National Institutes of Health; OR: Odds Ratio; CI: Confidence Interval; IQR: Interquartile Range; SD: Standard Deviation; SE: Standard Error; RR: Relative Risk; NA: not applicable.

## Data Availability

No new data were created or analyzed in this study. Data sharing is not applicable to this article.

## Supplementary Materials

The following supporting information can be downloaded at: https://www.mdpi.com/article/10.3390/muscles5040069/s1, Supplementary Material S1: Database-specific search strategies for PubMed, Scopus, and Web of Science.

## Abbreviations

The following abbreviations are used in this manuscript:

IF	Intrinsic factor
DXA	Dual-energy X-ray
MRI	Magnetic resonance imaging
CT	Computed Tomography
ROS	Reactive oxygen species
EFSA	European Food Safety Authority
WHO	World Health Organization
EWGSOP2	European Working Group on Sarcopenia in Older People
BIA	Bioelectrical Impedance Analysis
SPPB	Short Physical Performance Battery
TUG	Timed Up and Go test
Hcy	Homocysteine
MMA	Methylmalonic acid

## References

[1] Pyrgioti E.E., Karakousis N.D. (2022). B12 Levels and Frailty Syndrome. J. Frailty Sarcopenia Falls.

[2] Hannibal L., Lysne V., Bjørke-Monsen A.-L., Behringer S., Grünert S.C., Spiekerkoetter U., Jacobsen D.W., Blom H.J. (2016). Biomarkers and Algorithms for the Diagnosis of Vitamin B12 Deficiency. Front. Mol. Biosci..

[3] Mansoor U., Edano D., Usman M., Habib U. (2025). The Impact of Nutritional Supplements on Sarcopenia: A Systematic Review and Meta-Analysis. Cureus.

[4] Verlaan S., Aspray T.J., Bauer J.M., Cederholm T., Hemsworth J., Hill T.R., McPhee J.S., Piasecki M., Seal C., Sieber C.C. (2017). Nutritional Status, Body Composition, and Quality of Life in Community-Dwelling Sarcopenic and Non-Sarcopenic Older Adults: A Case-Control Study. Clin. Nutr..

[5] Ates Bulut E., Soysal P., Aydin A.E., Dokuzlar O., Kocyigit S.E., Isik A.T. (2017). Vitamin B12 Deficiency Might Be Related to Sarcopenia in Older Adults. Exp. Gerontol..

[6] Choi S., Chon J., Lee S.A., Yoo M.C., Chung S.J., Shim G.Y., Soh Y., Won C.W. (2023). Impact of Vitamin B12 Insufficiency on the Incidence of Sarcopenia in Korean Community-Dwelling Older Adults: A Two-Year Longitudinal Study. Nutrients.

[7] Baik H.W., Russell R.M. (1999). Vitamin B12 Deficiency in the Elderly. Annu. Rev. Nutr..

[8] Ströhle A., Richter M., González-Gross M., Neuhäuser-Berthold M., Wagner K.-H., Leschik-Bonnet E., Egert S., German Nutrition Society (DGE) (2019). The Revised D-A-CH-Reference Values for the Intake of Vitamin B12: Prevention of Deficiency and Beyond. Mol. Nutr. Food Res..

[9] Watanabe F. (2007). Vitamin B12 Sources and Bioavailability. Exp. Biol. Med..

[10] Obeid R., Heil S.G., Verhoeven M.M.A., van den Heuvel E.G.H.M., de Groot L.C.P.G.M., Eussen S.J.P.M. (2019). Vitamin B12 Intake From Animal Foods, Biomarkers, and Health Aspects. Front. Nutr..

[11] Fernandes S., Oliveira L., Pereira A., Costa M.D.C., Raposo A., Saraiva A., Magalhães B. (2024). Exploring Vitamin B12 Supplementation in the Vegan Population: A Scoping Review of the Evidence. Nutrients.

[12] De Jager J., Kooy A., Lehert P., Wulffele M.G., Van Der Kolk J., Bets D., Verburg J., Donker A.J.M., Stehouwer C.D.A. (2010). Long Term Treatment with Metformin in Patients with Type 2 Diabetes and Risk of Vitamin B-12 Deficiency: Randomised Placebo Controlled Trial. BMJ.

[13] Miller J.W. (2018). Proton Pump Inhibitors, H2-Receptor Antagonists, Metformin, and Vitamin B-12 Deficiency: Clinical Implications. Adv. Nutr..

[14] Guéant J.-L., Guéant-Rodriguez R.-M., Alpers D.H. (2022). Vitamin B12 Absorption and Malabsorption. Vitam. Horm..

[15] Pannérec A., Migliavacca E., De Castro A., Michaud J., Karaz S., Goulet L., Rezzi S., Ng T.P., Bosco N., Larbi A. (2018). Vitamin B12 Deficiency and Impaired Expression of Amnionless during Aging. J. Cachexia Sarcopenia Muscle.

[16] Cruz-Jentoft A.J., Bahat G., Bauer J., Boirie Y., Bruyère O., Cederholm T., Cooper C., Landi F., Rolland Y., Sayer A.A. (2019). Sarcopenia: Revised European Consensus on Definition and Diagnosis. Age Ageing.

[17] Voulgaridou G., Tyrovolas S., Detopoulou P., Tsoumana D., Drakaki M., Apostolou T., Chatziprodromidou I.P., Papandreou D., Giaginis C., Papadopoulou S.K. (2024). Diagnostic Criteria and Measurement Techniques of Sarcopenia: A Critical Evaluation of the Up-to-Date Evidence. Nutrients.

[18] Albano D., Messina C., Vitale J., Sconfienza L.M. (2020). Imaging of Sarcopenia: Old Evidence and New Insights. Eur. Radiol..

[19] Reiss J., Iglseder B., Kreutzer M., Weilbuchner I., Treschnitzer W., Kässmann H., Pirich C., Reiter R. (2016). Case Finding for Sarcopenia in Geriatric Inpatients: Performance of Bioimpedance Analysis in Comparison to Dual X-Ray Absorptiometry. BMC Geriatr..

[20] Lee R.C., Wang Z., Heo M., Ross R., Janssen I., Heymsfield S.B. (2000). Total-Body Skeletal Muscle Mass: Development and Cross-Validation of Anthropometric Prediction Models. Am. J. Clin. Nutr..

[21] Tseng T.-H., Fu S.-H., Sie N.-H., Lu Y.-C., Wang C.-Y., Wu C.-H. (2025). Epidemiology of Sarcopenia: A Narrative Review. Osteoporos. Sarcopenia.

[22] Papadopoulou S.K., Tsintavis P., Potsaki P., Papandreou D. (2020). Differences in the Prevalence of Sarcopenia in Community-Dwelling, Nursing Home and Hospitalized Individuals. A Systematic Review and Meta-Analysis. J. Nutr. Health Aging.

[23] Pacifico J., Geerlings M.A.J., Reijnierse E.M., Phassouliotis C., Lim W.K., Maier A.B. (2020). Prevalence of Sarcopenia as a Comorbid Disease: A Systematic Review and Meta-Analysis. Exp. Gerontol..

[24] Cannataro R., Carbone L., Petro J.L., Cione E., Vargas S., Angulo H., Forero D.A., Odriozola-Martínez A., Kreider R.B., Bonilla D.A. (2021). Sarcopenia: Etiology, Nutritional Approaches, and miRNAs. Int. J. Mol. Sci..

[25] Borba V.Z.C., Costa T.M.d.R.L. (2025). Sarcopenic Obesity: A Review. Arch. Endocrinol. Metab..

[26] Beaudart C., Alcazar J., Aprahamian I., Batsis J.A., Yamada Y., Prado C.M., Reginster J.-Y., Sanchez-Rodriguez D., Lim W.S., Sim M. (2025). Health Outcomes of Sarcopenia: A Consensus Report by the Outcome Working Group of the Global Leadership Initiative in Sarcopenia (GLIS). Aging Clin. Exp. Res..

[27] Beaudart C., Demonceau C., Reginster J.-Y., Locquet M., Cesari M., Cruz Jentoft A.J., Bruyère O. (2023). Sarcopenia and Health-Related Quality of Life: A Systematic Review and Meta-Analysis. J. Cachexia Sarcopenia Muscle.

[28] Zhang X., Zhang W., Wang C., Tao W., Dou Q., Yang Y. (2018). Sarcopenia as a Predictor of Hospitalization among Older People: A Systematic Review and Meta-Analysis. BMC Geriatr..

[29] Zhao Y., Jiang Y., Guo Y., Pan W., Tian W., Tang L., Feng X. (2025). Long-Term Impact of Sarcopenia on Functional Decline and Mortality in Community-Dwelling Older Adults: A Systematic Review and Meta-Analysis. Front. Nutr..

[30] Zhao J., Lu Q., Zhang X. (2024). Associations of Serum Vitamin B12 and Its Biomarkers with Musculoskeletal Health in Middle-Aged and Older Adults. Front. Endocrinol..

[31] Liu Y., Liu X., Duan L., Zhao Y., He Y., Li W., Cui J. (2024). Associations of Micronutrient Dietary Patterns with Sarcopenia among US Adults: A Population-Based Study. Front. Nutr..

[32] Chae S.A., Kim H.-S., Lee J.H., Yun D.H., Chon J., Yoo M.C., Yun Y., Yoo S.D., Kim D.H., Lee S.A. (2021). Impact of Vitamin B12 Insufficiency on Sarcopenia in Community-Dwelling Older Korean Adults. Int. J. Environ. Res. Public. Health.

[33] Soh Y., Won C.W. (2020). Association between Frailty and Vitamin B12 in the Older Korean Population. Medicine.

[34] Takahashi F., Hashimoto Y., Kaji A., Sakai R., Kawate Y., Okamura T., Kondo Y., Fukuda T., Kitagawa N., Okada H. (2021). Vitamin Intake and Loss of Muscle Mass in Older People with Type 2 Diabetes: A Prospective Study of the KAMOGAWA-DM Cohort. Nutrients.

[35] Borda M.G., Samuelsson J., Cederholm T., Baldera J.P., Pérez-Zepeda M.U., Barreto G.E., Zettergren A., Kern S., Rydén L., Gonzalez-Lara M. (2024). Nutrient Intake and Its Association with Appendicular Total Lean Mass and Muscle Function and Strength in Older Adults: A Population-Based Study. Nutrients.

[36] Polo-Ferrero L., Recio-Rodriguez J.I., González-Manzano S., Martín-Vallejo J., Barbero-Iglesias F.J., Montero-Errasquín B., Cruz-Jentoft A.J., Méndez-Sánchez R. (2025). Nutritional Intake as a Determinant of High-Speed Resistance and Multicomponent Training Efficacy on Strength in Older Women at Risk of Sarcopenia. A Randomized Clin. Trial. Clin. Nutr..

[37] Scott J., Yates M., Tanaka T., Ferrucci L., Cameron D., Welch A.A. (2025). Cross-Sectional Associations between Clinical Biochemistry and Nutritional Biomarkers and Sarcopenic Indices of Skeletal Muscle in the Baltimore Longitudinal Study of Aging. J. Nutr..

[38] Jia G., Wu C.-C., Su C.-H. (2023). Association between Dietary Antioxidant and Fatty Acids and Low Muscle Mass in Obese and Non-Obese Community-Dwelling Older Adults: An NHANES Analysis. Medicine.

[39] Wee A.K.H. (2016). Serum Folate Predicts Muscle Strength: A Pilot Cross-Sectional Study of the Association between Serum Vitamin Levels and Muscle Strength and Gait Measures in Patients > 65 Years Old with Diabetes Mellitus in a Primary Care Setting. Nutr. J..

[40] Ao M., Inuiya N., Ohta J., Kurose S., Takaoka H., Abe Y., Niki N., Inoue S., Tanaka S., Miyawaki T. (2019). Relationship between Homocysteine, Folate, Vitamin B12 and Physical Performance in the Institutionalized Elderly. J. Nutr. Sci. Vitaminol..

[41] van Schoor N.M., Swart K.M.A., Pluijm S.M.F., Visser M., Simsek S., Smulders Y., Lips P. (2012). Cross-Sectional and Longitudinal Association between Homocysteine, Vitamin B12 and Physical Performance in Older Persons. Eur. J. Clin. Nutr..

[42] Gedmantaite A., Celis-Morales C.A., Ho F., Pell J.P., Ratkevicius A., Gray S.R. (2020). Associations between Diet and Handgrip Strength: A Cross-Sectional Study from UK Biobank. Mech. Ageing Dev..

[43] Pedrero-Chamizo R., Albers U., Palacios G., Meléndez A., González-Gross M. (2020). Health Risk, Functional Markers and Cognitive Status in Institutionalized Older Adults: A Longitudinal Study. Int. J. Environ. Res. Public Health.

[44] Petermann-Rocha F., Chen M., Gray S.R., Ho F.K., Pell J.P., Celis-Morales C. (2020). Factors Associated with Sarcopenia: A Cross-Sectional Analysis Using UK Biobank. Maturitas.

[45] Wolffenbuttel B.H.R., Wouters H.J.C.M., de Jong W.H.A., Huls G., van der Klauw M.M. (2020). Association of Vitamin B12, Methylmalonic Acid, and Functional Parameters. Neth. J. Med..

[46] van Atteveld J.E., de Winter D.T.C., Pluimakers V.G., Fiocco M., Nievelstein R.A.J., Hobbelink M.G.G., Kremer L.C.M., Grootenhuis M.A., Maurice-Stam H., Tissing W.J.E. (2023). Frailty and Sarcopenia within the Earliest National Dutch Childhood Cancer Survivor Cohort (DCCSS-LATER): A Cross-Sectional Study. Lancet Healthy Longev..

[47] Sharma S., Bhadra R., Selvam S., Sambashivaiah S. (2024). Vitamin B12 Status and Skeletal Muscle Function among Elderly: A Literature Review and Pilot Study on the Effect of Oral Vitamin B12 Supplementation in Improving Muscle Function. Aging Med. Milton NSW.

[48] Qiu Y., Zhao F., Zheng X., Wang X. (2025). The Association between Dietary Vitamins and the Risk of Sarcopenia in Adults Aged 20–59: A Study Based on the NHANES Database. Front. Nutr..

