# Supplementary material for: Sarcopenia and Vitamin B12: Where Do We Stand?
Source: Muscles. 2026 Sep 29;5(4):69. doi: 10.3390/muscles5040069 (PMC13692420; doi:10.3390/muscles5040069)
Supplement: Supplementary file 1 [file muscles-05-00069-s001.zip › muscles-4426103-supplementary.pdf]

Supplementary Material 1

Database-Specific Search Strategy

A structured literature search was conducted in PubMed, Scopus, and Web of Science. The final search was performed on September 4, 2026. The search covered publications from January 1, 2010, to August 31, 2026. Search terms were adapted to the syntax and searchable fields of each database while maintaining the same conceptual search strategy across databases.

1. **PubMed**

Search string

("vitamin B12"[Title/Abstract] OR cobalamin[Title/Abstract])

AND

(

sarcopenia[Title/Abstract]

OR "low muscle mass"[Title/Abstract]

OR "low muscle strength"[Title/Abstract]

OR "muscle performance"[Title/Abstract]

)

Filters applied

Publication date: 2010/01/01–2026/09/4

Language: English

Species: Humans

Equivalent conceptual combinations

1. ("vitamin B12"[Title/Abstract] OR cobalamin[Title/Abstract]) AND sarcopenia[Title/Abstract]
2. ("vitamin B12"[Title/Abstract] OR cobalamin[Title/Abstract]) AND "low muscle mass"[Title/Abstract]
3. ("vitamin B12"[Title/Abstract] OR cobalamin[Title/Abstract]) AND "low muscle strength"[Title/Abstract]
4. ("vitamin B12"[Title/Abstract] OR cobalamin[Title/Abstract]) AND "muscle performance"[Title/Abstract]

2. **Scopus**

Search string

TITLE-ABS-KEY ("vitamin B12" OR cobalamin) AND TITLE-ABS-KEY (sarcopenia OR "low muscle mass" OR "low muscle strength" OR "muscle performance")

Filters applied

Publication year: 2010–2026

Language: English

Document type: Article

Equivalent conceptual combinations

1. TITLE-ABS-KEY ("vitamin B12" OR cobalamin) AND TITLE-ABS-KEY (sarcopenia)
2. TITLE-ABS-KEY ("vitamin B12" OR cobalamin) AND TITLE-ABS-KEY ("low muscle mass")
3. TITLE-ABS-KEY ("vitamin B12" OR cobalamin) AND TITLE-ABS-KEY ("low muscle strength")
4. TITLE-ABS-KEY ("vitamin B12" OR cobalamin) AND TITLE-ABS-KEY ("muscle performance")

---

3. **Web of Science**

Search string

TS=("vitamin B12" OR cobalamin) AND TS=(sarcopenia OR "low muscle mass" OR "low muscle strength" OR "muscle performance")

Filters applied

Publication years: 2010–2026

Language: English

Document type: Article

Equivalent conceptual combinations

1. TS=("vitamin B12" OR cobalamin) AND TS=(sarcopenia)
2. TS=("vitamin B12" OR cobalamin) AND TS=("low muscle mass")
3. TS=("vitamin B12" OR cobalamin) AND TS=("low muscle strength")
4. TS=("vitamin B12" OR cobalamin) AND TS=("muscle performance")

Study-selection criteria applied after database searching

Original human research articles published in English were considered eligible if they directly investigated vitamin B12 in relation to sarcopenia or one or more sarcopenia-related components, including muscle mass, muscle strength, or physical performance. Reviews, case reports, animal or in vitro studies, duplicate records, and studies not directly addressing the vitamin B12–sarcopenia relationship were excluded. Records remaining after database filtering and duplicate removal underwent title/abstract screening, followed by full-text eligibility assessment using the predefined inclusion and exclusion criteria.
